# Predictive approach for liberation from acute dialysis in ICU patients using interpretable machine learning

**DOI:** 10.1038/s41598-024-63992-y

**Published:** 2024-06-07

**Authors:** Tsai-Jung Wang, Chun-Te Huang, Chieh-Liang Wu, Cheng-Hsu Chen, Min-Shian Wang, Wen-Cheng Chao, Yi-Chia Huang, Kai-Chih Pai

**Affiliations:** 1 Department of Critical Care Medicine, Taichung Veterans General Hospital, Taichung, Taiwan, ROC; 2 Devision of Nephrology, Department of Internal Medicine, Taichung Veterans General Hospital, Taichung, Taiwan, ROC; 3 Department of Nutrition, Chung Shan Medical University, Taichung, Taiwan, ROC; 4 Department of Post-Baccalaureate Medicine, College of Medicine, National Chung Hsing University, Taichung, Taiwan, ROC; 5 Department of Nutrition, Chung Shan Medical University Hospital, Taichung, Taiwan, ROC; 6 College of Engineering, Tunghai University, No. 1727, Sec. 4, Taiwan Boulevard, Xitun District, Taichung City, 407224 Taiwan, ROC

**Keywords:** Acute kidney injury, Dialysis, Intensive care, Machine learning, Renal recovery, Medical research, Nephrology

## Abstract

Renal recovery following dialysis-requiring acute kidney injury (AKI-D) is a vital clinical outcome in critical care, yet it remains an understudied area. This retrospective cohort study, conducted in a medical center in Taiwan from 2015 to 2020, enrolled patients with AKI-D during intensive care unit stays. We aimed to develop and temporally test models for predicting dialysis liberation before hospital discharge using machine learning algorithms and explore early predictors. The dataset comprised 90 routinely collected variables within the first three days of dialysis initiation. Out of 1,381 patients who received acute dialysis, 27.3% experienced renal recovery. The cohort was divided into the training group (N = 1135) and temporal testing group (N = 251). The models demonstrated good performance, with an area under the receiver operating characteristic curve of 0.85 (95% CI, 0.81–0.88) and an area under the precision-recall curve of 0.69 (95% CI, 0.62–0.76) for the XGBoost model. Key predictors included urine volume, Charlson comorbidity index, vital sign derivatives (trend of respiratory rate and SpO2), and lactate levels. We successfully developed early prediction models for renal recovery by integrating early changes in vital signs and inputs/outputs, which have the potential to aid clinical decision-making in the ICU.

Acute kidney injury (AKI) and severe AKI that requires renal replacement therapy (RRT) initiation accounts for 50% and 5%–15% of intensive care units (ICU) patients, respectively^1,2^. AKI morbidity and mortality increase with increase in its severity and is highest among patients with AKI requiring dialysis (AKI-D)^3^. Outcomes for AKI-D can vary from successful recovery without the need for RRT to dialysis dependent, or even mortality^4^. Nonrecovery of renal function is a vital morbid event with long-term implications for both patients and healthcare system, including longer ICU stay, higher mortality, and economic burden^4^.

Over the past two decades, several studies have investigated various variables^5,6^, including conventional biochemical markers used as surrogates of kidney function^7,8^, urine output changes^9^, and novel kidney biomarkers^10,11^, to predict successful dialysis discontinuation. However, parameters assessed at the initiation or early stages of dialysis have shown relatively poor predictive values. Early and accurate stratification of renal prognosis is essential for closely monitoring renal function, avoiding nephrotoxic agents, applying kidney protective measures, adjusting dialysis parameters, and facilitating early and informed conversations regarding patient care goals^12^. Therefore, developing a precise prediction model shortly after the initiation of dialysis may enhance decision-making and care for patients with AKI-D in ICUs.

Predicting outcomes related to AKI holds significant clinical interest, yet challenging due to the complex and high-dimensional nature of ICU data. Machine learning prediction models have become vital tools, employed to forecast a variety of kidney-related outcomes. These models effectively identify patterns that not only predict the development and progression of AKI but also the potential need for acute dialysis and the long-term consequences following AKI^12–15^. However, to explore good machine-learning models for predicting successful dialysis liberation in patients with AKI-D remains an area of research^16^. To date, no widely accepted tool for early prediction of kidney recovery in patients with AKI-D exist^11^. Moreover, the absence of patient mortality as a competing risk remains a main concern in studies focusing on RRT liberation^12^. This study aimed to develop and validate clinically applicable machine-learning models for the early prediction of successful RRT discontinuation at discharge in patients with AKI-D admitted to ICU using routinely collected parameters obtained within the first 3 days after RRT initiation, as well as explore early predictors.

## Methods

### Study population

**Figure 1:**
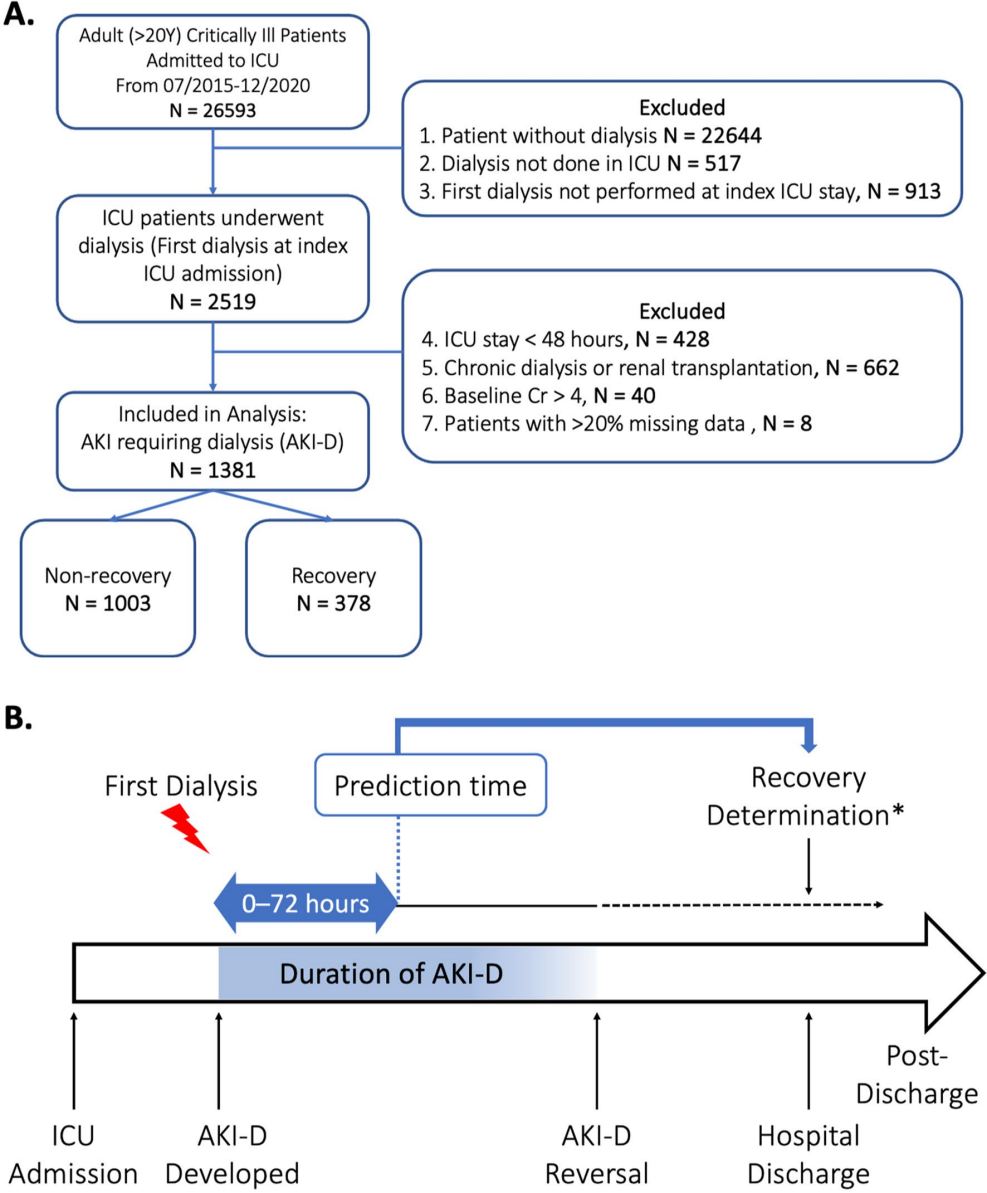
Overall schema of study. (**A**) Flowchart of the study population. (**B**) Illustration of the study design and the time frame: The blue bar with a double arrow represents the feature window for acute kidney injury requiring dialysis (AKI-D), which spanned the first 72 h following the commencement of dialysis. The prediction of recovery was assessed immediately after the end of this feature window. *Those recovered from AKI-D on discharge must fulfil two criteria: (i) being dialysis-free for at least a 5-day period before discharge; and (ii) being alive for more than 30 days after stopping dialysis.

Cases of AKI-D were defined as patients who experienced AKI and required RRT, excluding those with pre-existing end-stage renal disease (ESRD). We herein retrospectively identified adult patients who underwent their first dialysis due to AKI during the index ICU admission at Taichung Veterans General Hospital (TCVGH), a tertiary care teaching hospital in Taiwan with five adult ICUs and a computerized electronic medical record (EMR) system, between January 2015 and December 2020. The use of RRT was always reviewed and approved by nephrologists before application. We excluded patients who had been in the ICU for < 48 h, those with a history of chronic dialysis or renal transplant for ESRD, individuals with a baseline creatinine (Cr) ≥ 4 mg/dL, or those with > 20% missing data (Fig. 1A). This cohort was established from two databases: the clinical data warehouse at TCVGH and cause-of-death data from the National Health Insurance Research Database (NHIRD) in Taiwan to determine the date of death of the patients until the end of 2021.

### Data collection and feature selection

We identified candidate predictors from routinely collected data within 3 days after initiating dialysis: EMR data and time series of vital signs, which were categorized as follows: ‘Demographics and severity at admission’, variables such as the length of hospitalization before ICU admission and severity scores (the acute physiology and chronic health evaluation II [APACHE-II] score and the sequential organ failure assessment [SOFA]) were considered; ‘Comorbidities at admission’, determined using International Classification of Diseases, 9th Revision, Clinical Modification (ICD-9-CM) and 10th Revision (ICD-10) codes within the past year^17^; ‘Vital signs’, including vital sign measurements taken within 72 h after dialysis initiation; ‘Laboratory values’, including laboratory results obtained within the initial 72 h of dialysis; ‘Medications’, considering the use of potential nephrotoxic drugs over a 1-week period before the prediction time; ‘Inputs and outputs’, for fluid status data throughout the first 72 h of dialysis; and ‘AKI and dialysis parameters’, including the days from ICU admission to dialysis, AKI defined by either Cr or urine output criteria as outlined in the Kidney Disease: Improving Global Outcomes (KDIGO) clinical practice guidelines for AKI^18^, and dialysis mode. We assessed whether patients met the AKI diagnosis criteria before initiating dialysis: an increase in serum Cr of more than 0.3 mg/dL from baseline (label_Cr) and/or a decrease in urine output to less than 0.5 mL/kg/hour for more than 6 h (label_Ur). Furthermore, vital signs are recorded at least every two hours and inputs and outputs data every eight hours in the ICU. For instance, within a 72 h feature window, there are at least 36 entries of vital signs. Given the complexity of the vital signs measured over time, we calculated the mean, variance, and trend of these signs to capture the dynamics of patient physiology. Besides, each entry of diet, intravenous fluid, and urine output was standardized to an hourly rate to facilitate the calculation of trends. Data preprocessing details can be found in the supplemental material (Supplemental Table S1, S2, and Figure S1). Table 1 presents the final set of 90 predictors used in our models.

**Table 1:** List of predictors.

Categories (Number)	Descriptions (Feature window)	Features
Demographics and severity at admission (6)	On the first day of ICU admission	Age, medical admission, APACHE-II, ventilator, shock, days to ICU admission
Comorbidities at admission (18)	At admission	Myocardial infarction, congestive heart failure, peripheral vascular disease, cerebrovascular disease, dementia, chronic pulmonary disease, rheumatic disease, peptic ulcer disease, mild liver disease, diabetes without chronic complication, diabetes with chronic complication, hemiplegia or paraplegia, renal disease, any malignancy including lymphoma and leukaemia, moderate or severe liver disease, metastatic solid tumour, AIDS/HIV, Charlson comorbidity index
Vital signs (21)	Mean (72 h)	Systolic BP, diastolic BP, pulse pressure, oximetry, respiratory rate, pulse rate, body temperature
	Variance (72 h)	
	Trend (72 h)	
Laboratory values (11)	Mean (72 h)	Creatinine, WBC, Hb, platelet, BUN, albumin, total bilirubin, lactate, PHA, Na, blood culture*
Medications (18)	Record as user or non-use (1 week)	Renin-angiotensin system inhibitors, diuretics, PPI, H2 receptor antagonists, NSAID-COX I inhibitors, COX II inhibitors, vasopressin, norepinephrine, dopamine, epinephrine, dobutamine, vancomycin, bactrim, gentamicin, amikin, colistin, amphotericin B, total medications**
Inputs and outputs (12)	Sum (72 h)	Blood transfusion, diet, intravenous fluid, dialysis ultrafiltration, urine volume, fluid balance
	Trend (72 h)***	
AKI and dialysis parameters (4)	On the first day of dialysis	Days from ICU admission to dialysis, label_Cr****, label_Ur****, CRRT

AIDS/HIV, acquired immunodeficiency syndrome/human immunodeficiency virus, APACHE-II, acute physiology and chronic health evaluation II; BP, blood pressure; COX, cyclooxygenase; ICU, intensive care unit; NSAID, non-steroidal anti-inflammatory drug; PHA, pH in arterial blood gas; PPI: proton-pump inhibitor.

*We represented “blood culture” as a categorical variable during a 1-week time frame.

**Denotes the sum of categories of the 17 aforementioned drugs.

***Trend analysis was conducted exclusively for diet, intravenous fluid, and urine output.

**** Denotes whether patients met the acute kidney injury (AKI) diagnosis criteria for an increase in serum creatinine more than 0.3 mg/dL (label_Cr) and/or a decrease in urine output less than 0.5 mL/kg/hour for > 6 h (label_Ur) as determined by the Kidney Disease: Improving Global Outcomes (KDIGO) clinical practice guidelines for AKI right before the initiation of dialysis.

### Hyperparameter optimization

For the XGBoost model, we experimented with various hyperparameters, including the number of estimators (500, 1000), learning rates (0.01, 0.02, 0.1), and maximum depths (3, 5, 7, 10). Using the grid search method, we found that the optimal configuration was 500 estimators, a learning rate of 0.01, and a maximum depth of 5, which yielded the best performance results. Similarly, for the Random Forest model, we conducted grid search experiments with different numbers of trees (50, 100, 150, 200) and criteria (Gini, entropy) to determine the most effective parameters. The grid search identified 'Gini' as the best criterion and 100 trees as the optimal number of estimators for achieving superior performance in the Random Forest classifier.

### Outcome measure: renal recovery and patient survival

We annotated the outcome as recovery for patients with AKI-D who were both free from dialysis and survived beyond 30 days after discontinuing dialysis, defined as RRT cessation for at least 5 days before hospital discharge (Fig. 1B). To minimize potential misclassification of patients who discontinued dialysis due to withdrawal of care, the deceased date from NHIRD were also gathered to ensure that patients were alive for > 30 days after dialysis discontinuation.

For patients who still required dialysis but were alive upon hospital discharge, their status were checked based on the presence of dialysis-dependent catastrophic illness certificates, which confirmed true nonrecovery; these certificates are issued after a review by at least two nephrologists who carefully examine medical records^19^. Furthermore, we compared the all-cause mortality on the 90th day and 1 year after hospital discharge.

## Data analysis and model development

Patients were divided into two groups: the training group (those admitted between 2015 and 2019) and testing cohort (those admitted in 2020) (Supplemental Figure S1). The prediction time point was set at 72 h post the first dialysis commencement (Fig. 1B). During initial model development, the training dataset was randomly split into training and validation sets in an 80:20 ratio. A fivefold cross-validation was performed to avoid overfitting and considered a robust method for model evaluation prior to temporal testing. Dialysis cessation after AKI-D was predicted using several machine-learning algorithms: extreme gradient boosting (XGBoost), random forest (RF), and logistic regression (LR)^20–22^.

The area under receiver operating characteristic (AUROC) statistics along with sensitivity, specificity, Brier Score, accuracy, precision, recall, and F-1 score was used for the training and testing groups. In the testing datasets, the receiver operating characteristic (ROC) curve, precision-recall curve (PRC), calibration curve, and decision curve were referred to evaluate the predictive machine-learning models’ discrimination, accuracy, and clinical applicability.

### Sensitivity analysis

Additional analyses were conducted to validate the robustness and support the clinical utility of our study. First, we made predictions at various time points post-dialysis along with 72 h baseline models. Models were generated using data obtained during the first 24- or 48-h window after first dialysis (observation time window), respectively, to estimate the renal and patient outcomes earlier. The prediction of recovery was assessed immediately after the end of this feature window. Second, to construct more simple models, we applied Least Absolute Shrinkage and Selection Operator (LASSO) regression selection to generate a 24-variable out of 90-variable full model. Third, the number of true negative outcomes was higher than that of true positive outcomes, thus requiring class weighting to address data imbalance. The different thresholds were presented to examine the model prediction performance to determine an optimal threshold.

### Feature interpretation and statistical analysis

For the best-performing model (XGBoost), Shapley additive explanation (SHAP) values were employed to evaluate feature importance and their relationship with the outcome in the test set^23^. Partial dependence plots (PDP) were generated for the most influential variables, and individual feature interactions were assessed with the study outcome^24^. Additionally, we illustrated SHAP values and Local Interpretable Model-agnostic Explanations (LIME) to provide visual insights into the clinical utility of selected patients. Baseline characteristics between groups with different renal recovery statuses were examined for statistical significance using a threshold of *p* < 0.05. All analyses were performed using Python (version 3.7.6) and R programming (version 3.4.0).

### Ethical approval and consent to participate

The study was approved by the Institutional Review Board of Taichung Veterans General Hospital with a waiver of informed consent since this was a retrospective analysis of anonymous data (TCVGH IRB number: SE20249B and SE21098B).

## Results

### Clinical characteristics of the study participants

Overall, 26,593 adults were admitted to TCVGH ICUs during the study period. Among the eligible patients, 1,381 patients (61.9% male) experienced AKI-D during their ICU stays (Fig. 1A). The included patients had a median age of 68.0 (interquartile range, 58.0–79.0), with 89.4% requiring mechanical ventilation. Overall, 378 patients (27.3%) achieved renal recovery, whereas 1,003 patients experienced nonrecovery of renal function. Further, 660 patients (47.8%) passed away before dialysis discontinuation or after withdrawing dialysis, whereas 343 patients (24.8%) remained dependent on dialysis upon discharge. Table 2 summarizes baseline and clinical characteristics of patients with AKI-D, stratified by renal recovery status at hospital discharge. Of the recruited patients with AKI-D, 1,135 patients admitted between 2015 and 2019 constituted the training cohort, whereas 246 patients admitted in 2020 comprised the temporal testing cohort. Supplemental Table S3 provides the patient characteristics, stratified based on the train/test split. The train/test cohorts showed comparable age, Charlson comorbidity index (CCI), and patient outcomes.

**Table 2:** Characteristics of the 1,381 patients with acute kidney injury requiring dialysis categorized by renal recovery.

	All (N = 1,381)	Recovery (N = 378)	Nonrecovery (N = 1,003)	*p* value*
Basic characteristics				
Age, years	68.0 (58.0–79.0)	66.0(52.0–79.0)	69.0(59.0–79.0)	< 0.01
Male	855 (61.9%)	236 (62.4%)	619 (61.7%)	0.81
BMI, kg/m^2^	24.5 (21.4–28.0)	24.3 (21.1–27.4)	24.6 (21.6–28.1)	0.69
Comorbidities at admission				
Charlson comorbidity index	3 (2–5)	2 (1–4)	4 (2–6)	< 0.01
Renal disease	773 (56.0%)	170 (45.0%)	603 (60.1%)	< 0.01
Any malignancy	419 (30.1%)	79 (20.6%)	340 (33.6%)	< 0.01
Diabetes	570 (41.3%)	147 (38.9%)	423 (42.2%)	0.27
Congestive heart failure	360 (26.1%)	84 (22.2%)	276 (27.5%)	0.05
Coronary artery disease	269 (19.5%)	69 (18.3%)	200 (19.9%)	0.48
Hypertension	494 (35.8%)	117 (31%)	377 (37.6%)	0.02
Admission characteristics				
Medical ICU	993 (71.9%)	271 (71.7%)	722 (72.0%)	0.91
Emergent or scheduled surgery	177 (12.8%)	62 (16.4%)	115 (11.5%)	0.01
APACHE-II score	29 (24–33)	28.5 (22–32)	30 (24–34)	< 0.01
SOFA	11 (8–13)	10 (7–13)	11 (8–13)	< 0.01
Use of ventilator	1234 (89.4%)	329 (87.0%)	905 (90.2%)	0.09
Use of vasopressor	1060 (76.8%)	278 (73.5%)	782 (78.0%)	0.08
Vital signs				
Systolic BP (mmHg)	118.7 (103.0–138.0)	126.3 (110.8–141.7)	116.3 (99.3–135.2)	< 0.01
Diastolic BP (mmHg)	64.3 (54.3–75.0)	69.7 (59.7–79.0)	63.0 (52.2–73.3)	< 0.01
Pulse pressure (mmHg)	53.0 (40.0–67.7)	56.0 (42.7–69.9)	52.0 (38.7–66.8)	< 0.01
Oximetry (%)	97.7 (95.3–99.3)	98.7 (96.8–99.7)	97.3 (94.7–99.3)	< 0.01
Respiiratory rate	18.7 (16.0–22.7)	18.0 (16.0–20.3)	19.0 (16.0–23.7)	< 0.01
Pulse rate	86.7 (74.7–100.7)	86.2 (73.5–97.9)	87.3 (74.7–101.7)	0.28
Body temperature (℃)	36.3 (35.9–36.7)	36.4 (36.2–36.8)	36.3 (35.8–36.6)	< 0.01
Laboratory values				
WBC (10^3^/μL)	11.0 (7.5–16.6)	11.6 (7.9–15.8)	10.6 (7.4–16.9)	0.62
BUN (mg/dL)	56.0 (32.0–85.0)	51.5 (32.0–80.0)	58.0 (32.0–88.0)	0.04
Creatinine (mg/dL)	3.3 (1.9–5.1)	2.9 (1.6–4.6)	3.5 (2–5.4)	< 0.01
Lactate (mg/dL)	13.3 (8.3–25.1)	11.8 (8.3–17.8)	14.6 (8.3–32.4)	< 0.01
PHA	7.4 (7.4–7.4)	7.4 (7.4–7.5)	7.4 (7.3–7.4)	< 0.01
Patients status at dialysis initiation				
Days from ICU admission to dialysis (day)	1.4 (0.4–4.2)	1.1 (0.3–3.4)	1.6 (0.5–4.7)	< 0.01
AKI defined based by Cr (Label_Cr)	1107 (80.2%)	302 (79.9%)	805(80.3%)	0.88
AKI defined based by urine (Label_Ur)	1143 (82.8%)	272 (72.0%)	871 (86.8%)	< 0.01
Fluid balance since ICU admission (L)	4.9 (1.2–14.4)	4.4 (1.0–12.4)	5.2 (1.4–15.4)	0.06
CRRT	236 (17.1%)	58 (15.4%)	178 (17.8%)	0.29
Contributing factors of AKI				
Contrast medium	443 (32.1%)	128 (33.9%)	315 (31.4%)	0.38
Post-operative AKI	228 (16.5%)	75 (19.8%)	153 (15.3%)	0.04
Sepsis or septic shock	720 (52.1%)	198 (52.4%)	522 (52.0%)	0.91
Major bleeding/hypoperfusion	364 (26.4%)	97 (25.7%)	267 (26.6%)	0.72
Outcomes				
ICU stay, days	12.7 (6.1–23.1)	14.7 (7.8–26.0)	12.0 (5.6–22.2)	< 0.01
Hospital stay, days	25.0 (13.8–32.8)	30.5 (20.1–35.0)	22.6 (12.0–31.8)	< 0.01
90-day mortality	794 (57.5%)	50 (13.2%)	744 (74.2%)	< 0.01
1-year mortality	911 (66.0%)	113 (29.9%)	798 (79.6%)	< 0.01

Results displayed either as median (interquartile range) or count (%).

AKI, acute kidney injury; APACHE-II, acute physiology and chronic health evaluation II; BMI, body mass index; BP, blood pressure; CRRT, continuous renal replacement therapy; ICU, intensive care unit; PHA, pH in arterial blood gas; SOFA, sequential organ failure assessment.

* Chi-square test or Mann–Whitney U test as appropriate.

### Performance of the models

**Figure 2:**
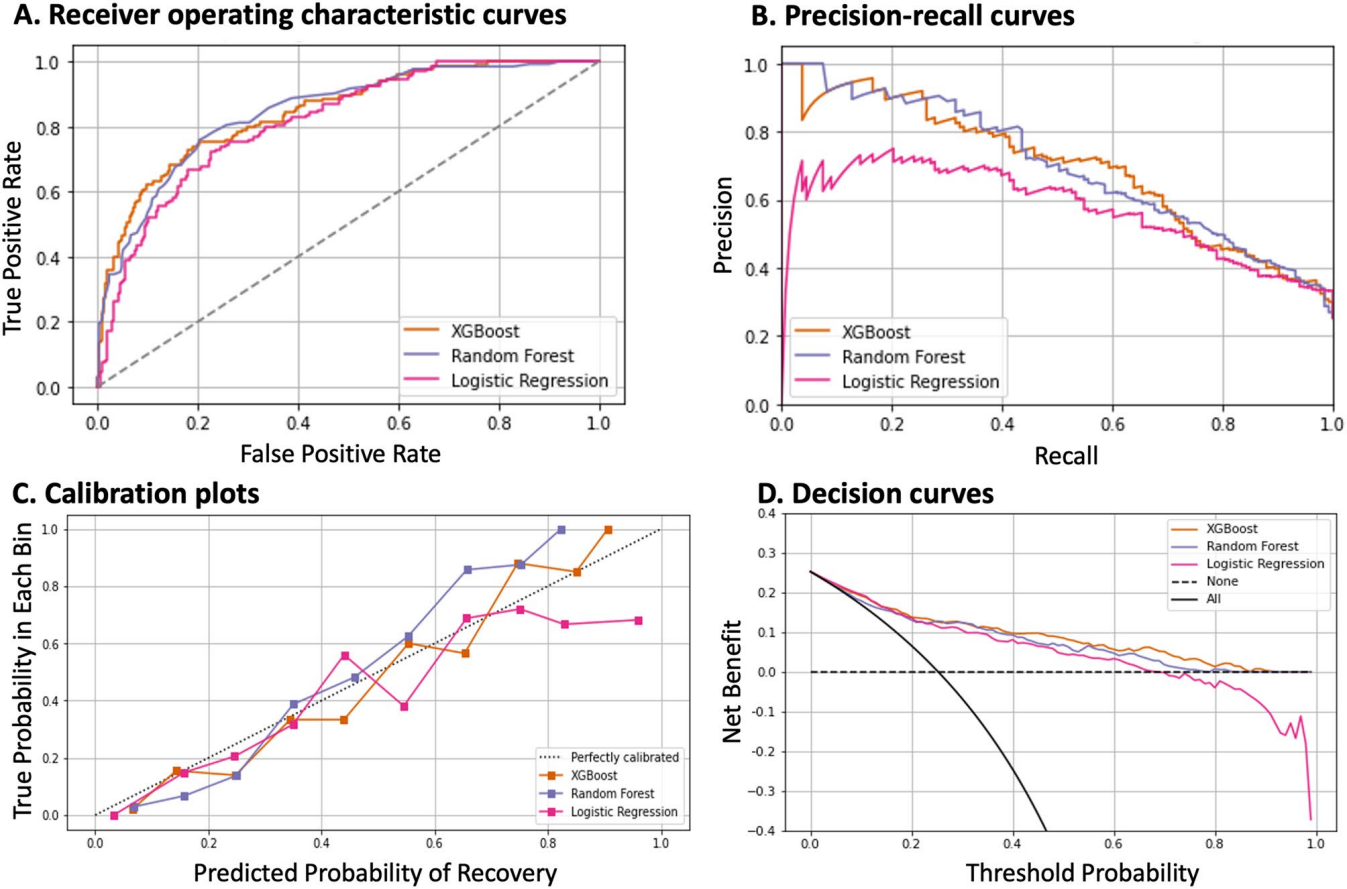
Performance of the machine-learning models for early prediction of dialysis liberation. (**A**) Area under receiver operating characteristic (AUROC) Plots: Area under curve of the temporal testing cohort, XGBoost 0.85 (95% CI, 0.81–0.88), RF 0.83 (95% CI, 0.80–0.87), and LR 0.82 (95% CI, 0.79–0.85). (**B**) Area under precision-recall curves (AUPRC), XGBoost 0.69 (95% CI, 0.62–0.76), RF 0.68 (95% CI, 0.62–0.76), and LR 0.58 (95% CI, 0.50–0.65). (**C**) Calibration plots. (**D**) Decision curves.

When trained and temporal tested with all 90 variables, the XGBoost, RF, and LR predictors achieved AUROC values of 0.85 (95% CI, 0.81–0.88), 0.83 (95% CI, 0.80–0.87), and 0.82 (95% CI, 0.79–0.85), respectively, for predicting renal recovery at 72 h windows in the testing cohort (Fig. 2A). XGBoost exhibited the highest accuracy among the three machine-learning models, and Delong’s test confirmed its significant outperformance of LR (Supplemental Table S4). Table 3 presents the performance statistics for the models with a proposed recovery threshold of 50%. Given this threshold examining the XGBoost model in the testing cohort, only 57% of the patients who were predicted to recover truly did recover (sensitivity), while 93% of the patients who were predicted not to recover indeed did not recover (specificity). Furthermore, the trade-off between sensitivity and specificity was evaluated at predicted renal recovery thresholds in increments of 10% for the XGBoost model (Supplemental Table S5). The F1 score, a good measure of test accuracy, was highest at a cut-off threshold of 0.3, while maintaining good values across the range of 0.2 to 0.5. Supplemental Table S6 demonstrates the performance matrix of a cut-off threshold of 0.3. At this threshold, the sensitivity increases to 73%, while the specificity is 81%. Additionally, The PRC for the models shown in Fig. 2B provided additional clarify on the model’s performance, especially for imbalance datasets, as in our study. Moreover, the temporal testing cohort had good calibration based on the disparity between the predicted and actual outcomes (Fig. 2C). Furthermore, the decision curve analysis illustrated favorable net benefits throughout a wide range of threshold probabilities (Fig. 2D).

**Table 3:** Performance of the machine-learning models for early prediction of dialysis liberation.

	Models	Sensitivity	Specificity	Brier Score	Accuracy	AUROC
**2015–5019** Development and fivefold cross-validation	XGBoost	0.48 ± 0.04	0.91 ± 0.04	0.22 ± 0.05	0.78 ± 0.05	0.81 ± 0.03
	RF	0.38 ± 0.02	0.95 ± 0.02	0.21 ± 0.03	0.79 ± 0.03	0.80 ± 0.02
	LR	0.43 ± 0.04	0.89 ± 0.04	0.24 ± 0.05	0.76 ± 0.05	0.77 ± 0.01
**2020** Temporal testing	XGBoost	0.57	0.93	0.16	0.84	0.85 (0.81–0.88)
	RF	0.44	0.94	0.18	0.82	0.83 (0.80–0.87)
	LR	0.44	0.91	0.21	0.79	0.82 (0.79–0.85)

AUROC, area under receiver operating characteristic; LR, logistic regression; RF, random forest; XGBoost, extreme gradient boosting. Values in parentheses are 95% confidence intervals.

### Independent predictors for renal recovery

**Figure 3:**
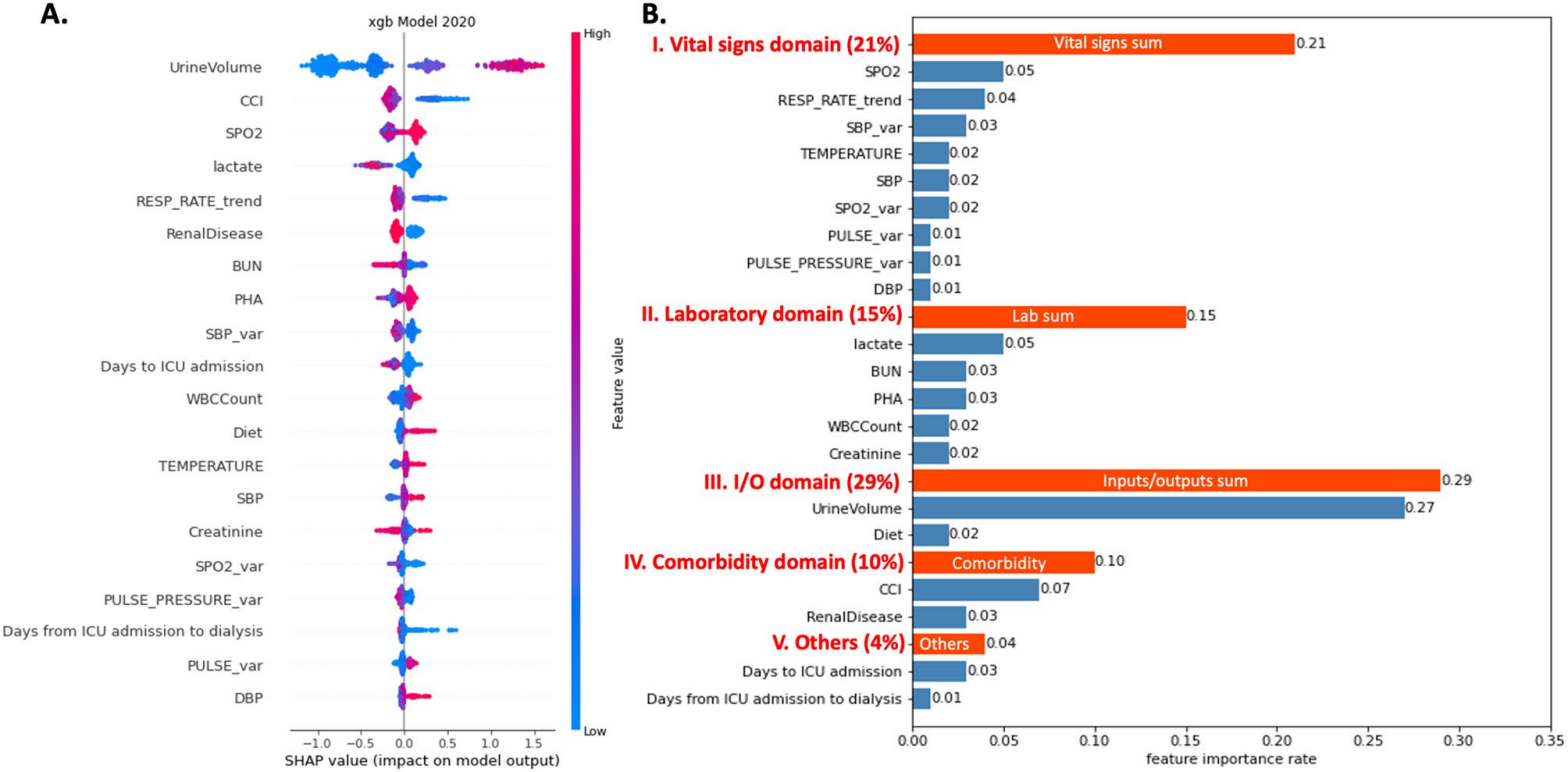
SHapley Additive exPlanation (SHAP) value to illustrate the renal recovery prediction model at feature level. (**A**) Top 20 SHAP summary plot: The features are listed from top to bottom based on their impact on the model prediction using SHAP values. (**B**) SHAP feature importance rate categorized by main clinical domains in predicting liberation of acute dialysis: The blue bars and numbers represent the proportion of each item in the model’s prediction. Cumulative feature importance by clinical domains is highlighted in red. Among these, the vital signs and inputs/outputs domains exhibit the highest level of contribution to the predictive model.

**Figure 4:**
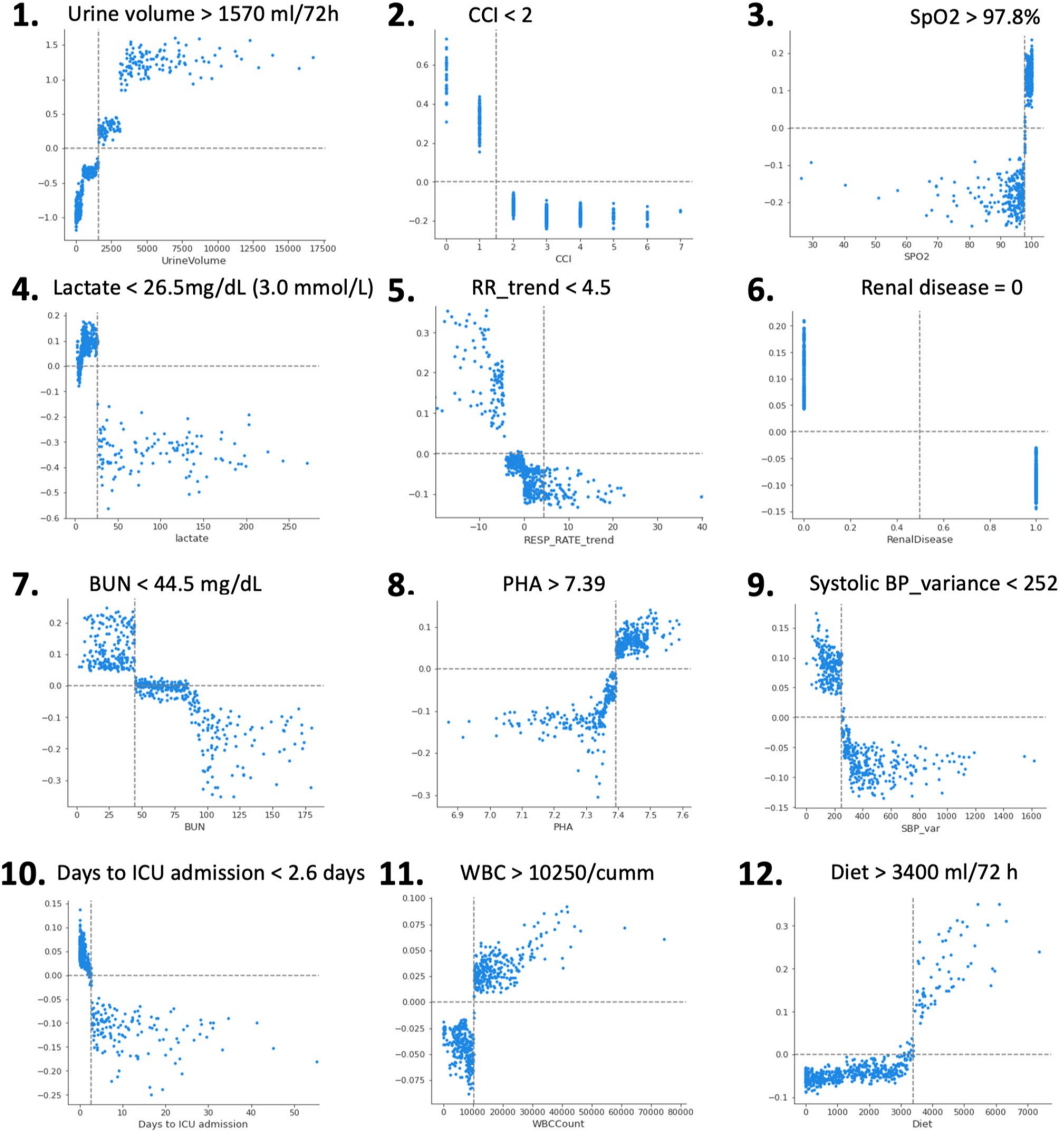
Partial dependence plots of the top 12 predictors by SHAP value in predicting renal recovery. The numbers indicate the order of importance according to SHAP values, and each plot includes an estimated cutoff value for the feature where the probability of renal recovery significantly increases. BP, blood pressure; CCI, Charlson Comorbidity Index; ICU, intensive care unit; PHA, arterial pH; RR, respiratory rate; SpO2, peripheral capillary oxygen saturation.

Figure 3A displays top 20 predictors of dialysis liberation and their XGBoost model rankings based on gain. Positive SHAP value (in red) suggests the feature boosts the predicted probability of dialysis liberation, while negative SHAP value (in blue) indicates a higher probability of dialysis dependency or death. The most influential feature was urine volume, followed by CCI, peripheral capillary oxygen saturation (SpO2), lactate, respiratory rate trend, renal disease, BUN, arterial PH, systolic blood pressure (BP) variation, days to ICU admission, white blood cell, and diet (Fig. 3). Figure 4 presents PDPs for those highest-ranking variables. Figure 3B demonstrates SHAP feature importance categorized by the main clinical domains in predicting dialysis liberation, highlighting the significant impact of early vital signs and inputs/outputs domains on the renal recovery probability.

### Sensitivity analyses

We conducted several additional analyses (Supplemental Table S7–S9). To address data imbalance (27.3% renal recovery vs. 72.7% nonrecovery), class weights were incorporated into the machine-learning models, resulting in consistent performance. Moreover, LASSO was used to select a reduced set of parameters for model building, retaining the top 24 most influential variables, with a c-statistic of 0.84 in the XGBoost model. Furthermore, dialysis liberation was predicted using different time windows: the original prediction at 72 h post-dialysis (Table 3) and earlier predictions at 48 and 24 h post-dialysis (Supplemental Table S9). These predictions yielded AUROC values of 0.84, 0.82, and 0.82, respectively, in the XGBoost model. Supplemental Figure S2 summarizes the ten most influential features in the model at 24, 48, and 72 h post-dialysis, along with their temporal evolution.

## Discussion

We constructed a machine-learning algorithm to forecast the early likelihood of dialysis liberation in critically ill patients with AKI, incorporating both the variability and trends of dynamic parameters from routine data within the first 72 h of dialysis. These models were developed, cross-validated, and temporal tested, thus demonstrating good discrimination in predicting renal recovery at hospital discharge. Furthermore, we applied class weighting to address data imbalance, used LASSO to develop models with few variables, and predicted short time windows (the first 24 or 48 h), all showing good discrimination. These results suggest the potential clinical utility of integration into EMR for clinical decision-support systems. Finally, using SHAP value and PDP, we identified critical features influencing the predictions of the model, with early vital signs and inputs/outputs domains being the vital drivers of the model. Explainable machine learning-based prediction for AKI-D recovery using existing EMR data hold potential for improving risk stratification and gaining insights into patient outcomes.

Nonrecovery of renal function after AKI-D is associated with increased morbidity and mortality and high health care cost^25,26^. Consistent with the findings of previous epidemiology studies^1,26^, the present study showed higher short-term (3-month) and long-term (1-year) post-discharge mortality rates for patients dependent on dialysis compared to those liberated from acute dialysis (Supplemental Figure S3). Early prediction of recovery from AKI-D in critically ill patients has significant implications regarding patient-centered care^27^. Currently, the prediction solely relies on clinical experience. The most commonly used dialysis cessation indicator is the increase in urinary output^5^. However, urinary output’s accuracy in predicting successful RRT discontinuation remains controversial, with reported AUROCs ranging from 0.63 to 0.91 and varying cut-off values^5,28^. Additionally, urinary output is typically employed as an indicator of renal recovery later upon RRT discontinuation rather than a marker in the early stages. Traditional functional biomarkers (serum/urine Cr or cystatin C) and novel biomarkers (kidney injury molecule-1, neutrophil gelatinase-associated lipocalin, osteopontin, tissue inhibitor of metalloprotease-2/insulin-like growth factor binding protein-7, proenkephalin A 119–159, etc.) have been explored as predictors of AKI-D recovery^5,8,11,27,29^. Current biomarkers for renal function recovery after AKI-D, which require additional samples and have limited conclusive evidence, have not been used widely to identify patients with a high probability of renal recovery in the early stages. The urgent need for precision guide to liberate from RRT was also recognized in the recent Acute Dialysis Quality Initiative (ADQI) consensus conference^30^. The experts emphasized on the integration of big data analysis and single case EMR evaluation to allow personalized RRT for every single individual. To address this gap, there is a clinical unmet need to integrate EMR to assess their predictive value for RRT discontinuation and prognosis in AKI-D.

Machine-learning models developed in critical nephrology can harness the data collected in EMR for important renal outcome predictions^13,31,32^. As data accumulates, these models will also offer the additional advantage of early prediction or enhanced accuracy. However, validated machine-learning models for predicting acute dialysis discontinuation in critical setting are less studied. To our knowledge, one prior research has employed a machine-learning approach to predict freedom from RRT in patients with AKI-D. Pattharanitima et al. utilized the Medical Information Mart for Intensive Care (MIMIC-III) database to predict RRT-free survival in critically ill patients with AKI requiring continuous renal replacement therapy (CRRT)^16^. Out of 684 patients, 30% had stopped from RRT successfully. Models using 81 features extracted between hospital admission and CRRT initiation yielded AUROC values ranging 0.43–0.7. In our study including 1,381 AKI-D individuals, we used 90 variables from the initial three days post-dialysis, including all vital signs and input–output records. Thus, variability and trends across multiple time points of these data were incorporated into our models. The prediction models in our study exhibited good performance, with AUROC of 0.77–0.81 in the development cohort and 0.82–0.85 in the temporal testing cohort. Aside from candidate predictors, the differences in model performance between our study and the prior study may also be due to differences in the study populations character, number of participants, and different feature window. The first three days are considered as acute phase of ICU patients, as exemplified by septic shock, where shock reversal often occurs within the first 3 days^33^. Importantly, providing additional prognostic information after initial intensive treatment period can aid in subsequent medical decisions, including the consideration of clinical trials for high-risk groups, or the potential withdrawal of life-sustaining medical care. Moreover, we trained models at 24 and 48 h in addition to the 72 h model, both maintaining good predictive performance.

As shown in Table 3 and Table S6, a proposed threshold of 0.5 for predicting renal recovery provided good specificity, whereas a threshold of 0.3 enhanced sensitivity. Decision curve analysis revealed the net benefits of using these models in clinical decision-making by considering the trade-offs between sensitivity and specificity at various threshold probabilities. The model’s use would yield more benefit than harm at both threshold of 0.5 and 0.3. Consequently, a lower threshold, such as 0.3, allowed for the identification of a broader subset of patients likely to recover renal function. Meanwhile, a threshold of 0.5 resulted in fewer false positives and would reduce alarm fatigue, a major concern in ICU alarm systems^34,35^. Therefore, the selection of a threshold in practical applications should be based on whether a healthcare provider requires assistance in accurately identifying patients who can recover or cannot recover after AKI-D, while effectively managing resources.

Using the interpretable machine-learning algorithm, nonsurprisingly, the single most influential variables in renal function recovery after initiating dialysis for AKI was urine output. Patients who successfully liberated from RRT demonstrated significantly higher urine output. According to PDP (Fig. 4), patients with urine volume > 1570 ml over the 72 h period post-dialysis were more likely to achieve dialysis independence at discharge. Figure 4 demonstrates the PDP of top predictors by SHAP value and the cut-off value in favor of renal recovery. In our study, the top 20 variables include previously well-studies factors for renal recovery, including urine volume or BUN^5,36,37^, along with less-explored variables (ex: enteral diet intake within the first 3 days after dialysis). Moreover, we categorized the top 20 variables identified by the XGBoost model by clinical domains, including comorbidity, vital signs, laboratory data, Inputs/outputs domain, and others. Besides urine volume, most of the early predictors were related to the vital signs domain (Fig. 3B). A general consensus exists that hemodynamic instability caused by excessive fluid removal during dialysis hinders renal recovery^38^. However, traditional prediction models for renal recovery have often overlooked vital signs due to their complexity and dynamic nature. Bellomo et al. conducted a retrospective study of critically ill patients with shock and found that higher levels of relative hypotension during the first few hours of vasopressor support were significantly associated with an increased risk of adverse kidney-related outcomes^39^. In line with the current evidence, our data suggests that early vital signs, not only the variance of systolic BP, but also SpO2, trend of respiratory rate, were significantly associated with the renal prognosis of critically ill patients. Additionally, the use of LASSO model with more limited variables and the incorporation of routinely collected laboratory data offer a practical means of rapid integration into EMR (Supplemental Table S8). An illustrate the interpretability of the models and the evolving of the key features over time using two separate individuals is presented in Supplemental Figure S4. Altogether, explainable machine-learning models can be deconvoluted to unveil new insights of how ICU patient features at the early stage interact with patient future events.

Strengths of our study include its size, comprising 1,381 patients with AKI-D among 26,593 ICU admissions. We also have complete data on vital signs and inputs/outputs with very low missing rates (< 1%). Furthermore, we linked the NHIRD cause-of-death data to mitigate withdrawal bias risk. This is especially important in ICU studies, as 40%–60% of critically ill patients with AKI-D have their treatment discontinued due to life support withdrawal or death^4^.

Our study has several limitations. First, recovery status was determined at hospital discharge; however, we recognized that dialysis liberation may proceed further. Nevertheless, the median hospital stay of critically ill patients with AKI-D was long (25.0 days for the entire cohort and 30.5 days for the recovery group), with dialysis-dependent catastrophic illness certificates verified before discharge by nephrologist among AKI-D nonrecovers. Thus, the kidney prognosis is clinically relevant. Second, our models made one-time early predictions of renal recovery on the basis of data obtained within 3 days of dialysis initiation. Events after that may drive the patient outcome away from the predictions. Though, we developed additional models at various time horizons (1 and 2 days), a continuously updating prediction is more appropriate for such cases. Third, limitations of our retrospective database include lack of other predictors of interest, including the degree of urine proteinuria, timed creatinine clearance, and novel kidney biomarkers, which may influence renal and patient recovery. Last, we used temporal testing, which is considered as an in-between validation of internal and external validation^40^. Although the recovery and mortality rates in this cohort was comparable to those reported in the literature^4,26^, the results should be further validated in other settings.

## Conlusions

To conclude, we developed, validated, and temporal tested applicable machine-learning prediction models using routinely collected clinical and laboratory data during the initial 72 h of dialysis to identify successful RRT discontinuation on hospital discharge in critically ill patients with AKI-D. Early vital signs and urine output are vital factors influencing the model. The interpretable machine learning-based algorithm can be used to facilitate patient-centered care in case of a complex scenario and vulnerable populations such as those with AKI-D. Models should be further validated and implemented in critically ill patients.

### Supplementary Information

Supplementary Information.

## Data Availability

The data is available from the corresponding author on reasonable request.

## Abbreviations

ADQI: Acute Dialysis Quality Initiative
AKI: Acute kidney injury
AKI-D: AKI requiring dialysis
APACHE-II: Acute physiology and chronic health evaluation II
AUPRC: Area under precision-recall curve
AUROC: Area under receiver operating characteristic
BP: Blood pressure
CCI: Charlson comorbidity index
Cr: Creatinine
CRRT: Continuous renal replacement therapy
EMR: Electronic medical record
ESRD: End-stage renal disease
ICU: Intensive care unit
KDIGO: The Kidney Disease: Improving Global Outcomes
LASSO: Least Absolute Shrinkage and Selection Operator
LIME: Local Interpretable Model-agnostic Explanations
LR: Logistic regression
MIMIC-III: Medical Information Mart for Intensive Care
NHIRD: National Health Insurance Research Database
PDP: Partial dependence plots
PRC: Precision-recall curve
RF: Random forest
RRT: Renal replacement therapy
SHAP: Shapley additive explanation
SOFA: Sequential organ failure assessment
SpO2: Saturation of peripheral oxygen
TCVGH: Taichung Veterans General Hospital
XGBoost: Extreme gradient boosting
