# Supplementary material for: Predictive approach for liberation from acute dialysis in ICU patients using interpretable machine learning
Source: Sci Rep. 2024 Jun 7;14:13142. doi: 10.1038/s41598-024-63992-y (PMC11161460; doi:10.1038/s41598-024-63992-y)

**Supplementary File**

**Table S1.** Plausible range for clinical and laboratory values

| **Variables** | **Plausible range** | **Unit** |
| --- | --- | --- |
| **Age** | 20–100 | years |
| **Systolic BP** | 0–300 | mmHg |
| **Diastolic BP** | 0–150 | mmHg |
| **Pulse pressure** | 0–200 | mmHg |
| **Oximetry** | 0–100 | % |
| **Respiratory rate** | 0–50 | Breath/min |
| **Pulse rate** | 0–300 | Beat/min |
| **Body temperature** | 25–45 | °C |
| **Urine volume** | 0–5,000 | ml |
| **Intravenous fluid** | 0–10,000 | ml |
| **Diet amount** | 0–3,000 | ml |
| BP, blood pressure. | | |

**Table S2.** An overview of the degree of missingness for the candidate features

| **Categories** | **Candidate Features** | **Missing rate (72 hours)** |
| --- | --- | --- |
| **Demographics and**  **severity at admission** | APACHE-II Score | 16.08% |
|  | SOFA^*^ | 21.80% |
| **Vital signs** | Systolic BP | 0.07% |
|  | Diastolic BP | 0.07% |
|  | Pulse Pressure | 0.07% |
|  | Oximetry | 1.52% |
|  | Respiratory rate | 0.07% |
|  | Pulse rate | 0.07% |
|  | Body temperature | 0.51% |
| **Laboratory values** | WBC | 2.90% |
|  | Neut^*^ | 23.75% |
|  | Hb | 2.90% |
|  | Platelet count | 2.90% |
|  | BUN | 4.49% |
|  | Cr | 3.11% |
|  | Albumin | 12.74% |
|  | Total bilirubin | 5.07% |
|  | Lactate | 11.66% |
|  | PHA | 8.76% |
|  | Glucose^*^ | 37.65% |
|  | Na | 2.82% |
|  | CRP^*^ | 46.42% |

^*^ Excluded features with >20% missing data from entire cohort.

APACHE-II, acute physiology and chronic health evaluation II; BP, blood pressure; Neut, the percentage of neutrophils among white blood cells; PHA, arterial pH; BP, blood pressure.

**Figure S1. Overview of the analytic pipeline.** We established upper and lower bounds for clinical and laboratory parameters to exclude outliers. In handling missing data, we first removed features with over 20% missing data across patients. Subsequently, we excluded patients with over 20% missing data across the remaining features. For missing data, we imputed values randomly within the normal range.

**
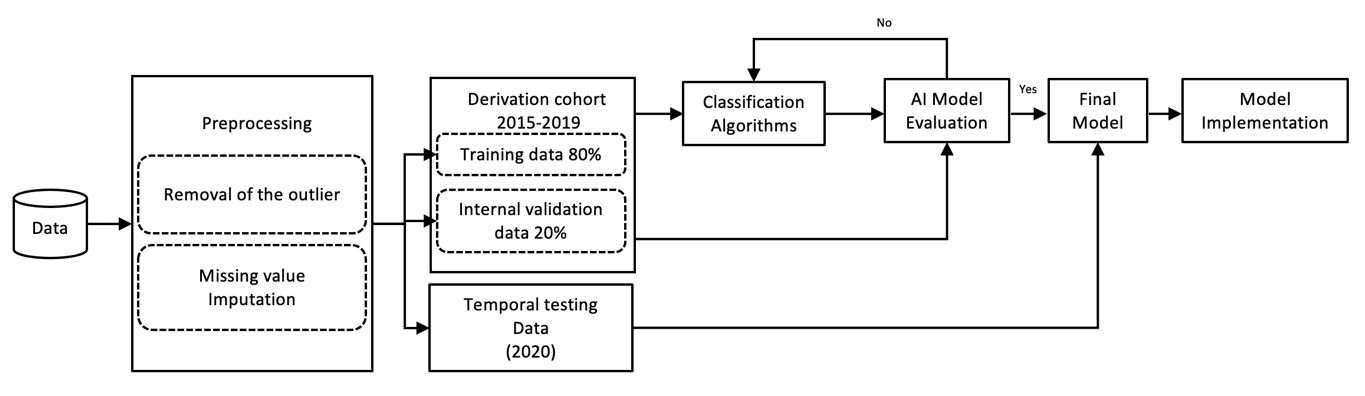
**

**Calculation of Temporal Features**

Depending on the model—whether it's a 24-, 48-, or 72-hour model—we select and analyze all data within the respective feature window.

**Variance:** In our analysis, each vital sign variable, such as systolic blood pressure, is observed as a series of data points throughout the chosen feature window. We calculate the variance using the sample variance formula to quantify the variability or dispersion of these measurements. A smaller variance indicates that the data points are closely clustered around the mean, while a larger variance suggests greater spread within the data.

**Trend:** To identify trends in time-series data, including vital signs, diet, intravenous fluid, and urine volume, we use polynomial regression. This method fits a non-linear model to the data, capturing complex behaviors over time more effectively than a simple linear trend. Specifically, we apply the polynomial fitting function **p = polyfit(x, y, n)**, where **x** and **y** are the data points, and **n** is the degree of the polynomial. The resulting polynomial 𝑝(𝑥)=𝑝1𝑥𝑛+𝑝2𝑥𝑛−1+...+𝑝𝑛𝑥+𝑝𝑛+1*p*(*x*)=*p*1​*xn*+*p*2​*xn*−1+...+*pn*​*x*+*pn*+1​ is the best fit for the data in a least-squares sense. The coefficients of this polynomial describe the trend of the variables, with higher-degree polynomials capturing more fluctuations.

This methodology allows us to extract meaningful features from the physiological data collected, providing deeper insights into patient conditions over time.

**Table S3.** Characteristics of the training (2015–2019) and temporal testing (2020) cohorts

|  | **Training corhort**  **(2015**–**2019)**  **(N = 1,135)** | **Temporal testing corhort (2020)**  **(N = 246)** | ***p* value^*^** |
| --- | --- | --- | --- |
| **Basic characteristics** |  | | |
| Age, years | 68.0 (58.0–79.0) | 68.5 (59.0–78.0) | 0.75 |
| Male | 705 (62.1%) | 150 (61.0%) | 0.74 |
| BMI, kg/m^2^ | 24.5 (21.4–28.0) | 24.4 (22.0–28.1) | 0.19 |
| **Comorbidities at admission** |  |  |  |
| Charlson Comorbidity Index | 3.0 (2.0–5.0) | 3.0 (2.0–5.0) | 0.98 |
| Chronic kidney disease | 625 (55.1%) | 148 (60.2%) | 0.14 |
| Any malignancy | 344 (30.3%) | 75 (30.5%) | 0.96 |
| Diabetes | 464 (40.9%) | 106 (43.1%) | 0.52 |
| Congestive heart failure | 297 (26.2%) | 63 (25.6%) | 0.86 |
| Coronary artery disease | 207 (18.2%) | 62 (25.2%) | 0.01 |
| Coronary artery disease | 397 (34.978%) | 97 (39.4309%) | 0.19 |
| **Admission characteristics** |  |  |  |
| Medical ICU | 822 (72.4%) | 194 (78.9%) | 0.04 |
| Emergent or scheduled surgery | 152 (13.4%) | 25 (10.2%) | 0.17 |
| APACHE-II score | 30.0 (24.0–34.0) | 28.0 (23.0–32.0) | 0.05 |
| SOFA | 10.0 (8.0–13.0) | 11.0 (8.0–13.0) | 0.26 |
| Use of Ventilator | 717 (63.2%) | 147 (59.8%) | 0.32 |
| Use of vasopressor | 1056 (93.0%) | 224 (91.1%) | 0.28 |
| **Patients status at dialysis initiation** |  |  |  |
| Days from ICU admission to dialysis (day) | 1.4 (0.4–4.4) | 1.2 (0.3–3.7) | 0.13 |
| AKI defined based by Cr (Label_Cr) | 903 (79.6%) | 204 (82.9%) | 0.23 |
| AKI defined based by urine (Label_Ur) | 929 (81.9%) | 214 (87.0%) | 0.05 |
| Serum creatinine (mg/dL) | 3.2 (1.7–5.0) | 3.3 (2.0–5.2) | 0.63 |
| Fluid balance since ICU admission (L) | 5.0 (1.4–14.3) | 4.5 (1.0–14.2) | 0.42 |
| CRRT | 187 (16.5%) | 49 (20.2%) | 0.17 |
| **Contributing factors of AKI** |  | | |
| Contrast medium | 350 (30.8%) | 93 (37.8%) | 0.03 |
| Post-operative AKI | 188 (16.6%) | 40 (16.3%) | 0.91 |
| Sepsis or septic shock | 608 (53.6%) | 112 (45.5%) | 0.02 |
| Major bleeding/hypoperfusion | 323 (28.5%) | 41 (16.7%) | <0.01 |
| **Outcomes** |  |  |  |
| ICU-stay, days | 12.6 (6.0–23.7) | 13.2 (6.6–21.9) | 0.76 |
| Hospital-stay, days | 24.9 (13.7–32.8) | 26.6 (14.1–32.2) | 0.65 |
| 90-day mortality | 658 (58.0%) | 136 (55.3%) | 0.44 |
| 1-year mortality | 753 (66.3%) | 158 (64.2%) | 0.53 |

Results displayed either as median (interquartile range) or count (%).

AKI, acute kidney injury; APACHE-II, acute physiology and chronic health evaluation II; BMI, body mass index; CRRT, continuous renal replacement therapy; ICU, intensive care unit; SOFA, sequential organ failure assessment.

* Chi-square test or Mann-Whitney U test as appropriate

**Table S4.** Delong’s test to determine the differences in performances among three machine-learning models

|  | **XGBoost** | **Random forest** | **Logistic regression** |
| --- | --- | --- | --- |
| **XGBoost** | NA | 0.73 | 0.04 |
| **Random forest** | 0.74 | NA | 0.06 |
| **Logistic regression** | 0.04 | 0.06 | NA |

LR, logistic regression; RF, random forest; XGBoost, extreme gradient boosting

**Table S5.** Predicted probability of renal recovery on discharge among critically ill patients requiring acute dialysis across 10% through 90% thresholds in the XGBoost model

|  | **Threshold** | **Sensitivity** | **Specificity** | **F-score** | **TN** | **FP** | **FN** | **TP** |
| --- | --- | --- | --- | --- | --- | --- | --- | --- |
| **Validation**  **(2015**–**2019)** | 0.1 | 0.98 ± 0.02 | 0.26 ± 0.03 | 0.51 ± 0.05 | 156 | 454 | 6 | 239 |
|  | 0.2 | 0.84 ± 0.03 | 0.59 ± 0.03 | 0.59 ± 0.04 | 362 | 248 | 40 | 205 |
|  | **0.3** | **0.69 ± 0.04** | **0.76 ± 0.04** | **0.60 ± 0.04** | **467** | **143** | **76** | **169** |
|  | 0.4 | 0.56 ± 0.02 | 0.85 ± 0.05 | 0.59 ± 0.03 | 523 | 87 | 108 | 137 |
|  | **0.5** | **0.48 ± 0.04** | **0.91 ± 0.04** | **0.56 ± 0.05** | **554** | **56** | **128** | **117** |
|  | 0.6 | 0.35 ± 0.04 | 0.96 ± 0.02 | 0.48 ± 0.03 | 584 | 26 | 159 | 86 |
|  | 0.7 | 0.20 ± 0.06 | 0.98 ± 0.01 | 0.32 ± 0.09 | 598 | 12 | 195 | 50 |
|  | 0.8 | 0.08 ± 0.03 | 1.0 ± 0.0 | 0.16 ± 0.05 | 609 | 1 | 224 | 21 |
|  | 0.9 | 0.00 ± 0.01 | 1.0 ± 0.0 | 0.03 | 610 | 0 | 244 | 1 |
| **Testing**  **(2020)** | 0.1 | 0.98 | 0.34 | 0.5 | 132 | 261 | 2 | 131 |
|  | 0.2 | 0.81 | 0.64 | 0.56 | 250 | 143 | 25 | 108 |
|  | **0.3** | **0.73** | **0.81** | **0.63** | **317** | **76** | **36** | **97** |
|  | 0.4 | 0.65 | 0.87 | 0.63 | 341 | 52 | 47 | 86 |
|  | **0.5** | **0.57** | **0.93** | **0.64** | **364** | **29** | **57** | **76** |
|  | 0.6 | 0.41 | 0.96 | 0.54 | 377 | 16 | 78 | 55 |
|  | 0.7 | 0.32 | 0.98 | 0.46 | 386 | 7 | 91 | 42 |
|  | 0.8 | 0.14 | 0.99 | 0.23 | 390 | 3 | 115 | 18 |
|  | 0.9 | 0.01 | 1 | 0.01 | 393 | 0 | 132 | 1 |

FN, false negative; FP, false positive; TN, true negative; TP, true positive

**Table S6.** Performance of the three machine learning models for early prediction of liberation from dialysis in ICU at cut-off threshold of 0.3

|  | **Models** | **Sensitivity** | **Specificity** | **Brier Score** | **Accuracy** | **AUROC** |
| --- | --- | --- | --- | --- | --- | --- |
| **2015**–**5019**  **Development and 5-fold cross validation** | XGBoost | 0.69 ± 0.04 | 0.76 ± 0.04 | 0.26 ± 0.02 | 0.74 ± 0.02 | 0.81 ± 0.03 |
|  | RF | 0.74 ± 0.05 | 0.7 ± 0.05 | 0.29 ± 0.04 | 0.71 ± 0.04 | 0.80 ± 0.02 |
|  | LR | 0.67 ± 0.03 | 0.74 ± 0.02 | 0.28 ± 0.02 | 0.72 ± 0.02 | 0.77 ± 0.01 |
| **2020**  **Temporal testing** | XGBoost | 0.73 | 0.81 | 0.21 | 0.79 | 0.85 (0.81–0.88) |
|  | RF | 0.78 | 0.72 | 0.26 | 0.74 | 0.83 (0.80–0.87) |
|  | LR | 0.72 | 0.77 | 0.25 | 0.75 | 0.82 (0.79–0.85) |

AUROC, Area under receiver operating characteristic; ICU, intensive care unit; LR, logistic regression; RF, random forest; XGBoost, extreme gradient boosting. Values in parentheses are 95% confidence intervals.

**Table S7.** Machine learning algorithm performance in predicting dialysis liberation before patient discharge with class weights

|  | **Models** | **Sensitivity** | **Specificity** | **Brier Score** | **Accuracy** | **AUROC** |
| --- | --- | --- | --- | --- | --- | --- |
| **2015–5019**  **Development and 5-fold cross validation** | XGBoost | 0.65 ± 0.06 | 0.82 ± 0.04 | 0.22 ± 0.03 | 0.78 ± 0.03 | 0.82 ± 0.02 |
|  | RF | 0.74 ± 0.11 | 0.716 ± 0.05 | 0.27 ± 0.03 | 0.73 ± 0.03 | 0.82 ± 0.02 |
|  | LR | 0.70 ± 0.06 | 0.73 ± 0.07 | 0.27 ± 0.06 | 0.72 ± 0.06 | 0.78 ± 0.04 |
| **2020**  **Temporal testing** | XGBoost | 0.69 | 0.83 | 0.20 | 0.79 | 0.84 (0.82–0.88) |
|  | RF | 0.76 | 0.77 | 0.23 | 0.77 | 0.84 (0.82–0.88) |
|  | LR | 0.72 | 0.74 | 0.25 | 0.74 | 0.81 (0.79–0.85) |

AUROC: Area under receiver operating characteristic; LR, logistic regression; RF, random forest; XGBoost, extreme gradient boosting. Values in parentheses are 95% confidence intervals.

**Table S8.** Machine learning algorithm performance with 24-variable LASSO models in early prediction of dialysis liberation before patient discharge

|  | **Models** | **Sensitivity** | **Specificity** | **Brier Score** | **Accuracy** | **AUROC** |
| --- | --- | --- | --- | --- | --- | --- |
| **2015–5019**  **Development and 5-fold cross validation** | XGBoost | 0.47 ± 0.07 | 0.92 ± 0.05 | 0.21 ± 0.04 | 0.79 ± 0.04 | 0.81 ± 0.05 |
|  | RF | 0.43 ± 0.06 | 0.91 ± 0.06 | 0.23 ± 0.05 | 0.78 ± 0.05 | 0.80 ± 0.05 |
|  | LR | 0.44 ± 0.06 | 0.91 ± 0.03 | 0.22 ± 0.04 | 0.78 ± 0.04 | 0.80 ± 0.04 |
| **2020**  **Temporal testing** | XGBoost | 0.56 | 0.97 | 0.17 | 0.83 | 0.84 (0.82–0.88) |
|  | RF | 0.50 | 0.92 | 0.18 | 0.82 | 0.83 (0.80–0.87) |
|  | LR | 0.45 | 0.92 | 0.20 | 0.80 | 0.83 (0.80–0.86) |

AUROC, Area under receiver operating characteristic; LASSO, Least Absolute Shrinkage and Selection Operator; LR, logistic regression; RF, random forest; XGBoost, extreme gradient boosting. Values in parentheses are 95% confidence intervals.

**Table S9.** Machine learning algorithm performance in predicting dialysis liberation before patient discharge at (A) 24 hours, and (B) 48 hours with class weights

**A.**

| **24 hours** | **Models** | **Sensitivity** | **Specificity** | **Brier Score** | **Accuracy** | **AUROC** |
| --- | --- | --- | --- | --- | --- | --- |
| **2015–5019**  **Development and 5-fold cross validation** | XGBoost | 0.63 ± 0.11 | 0.82 ± 0.03 | 0.24 ± 0.02 | 0.77 ± 0.02 | 0.78 ± 0.03 |
|  | RF | 0.73 ± 0.07 | 0.69 ± 0.04 | 0.29 ± 0.03 | 0.70 ± 0.03 | 0.79 ± 0.04 |
|  | LR | 0.62 ± 0.09 | 0.71 ± 0.05 | 0.32 ± 0.05 | 0.68 ± 0.05 | 0.73 ± 0.06 |
| **2020**  **Temporal testing** | XGBoost | 0.69 | 0.74 | 0.27 | 0.73 | 0.78 (0.75–0.82) |
|  | RF | 0.74 | 0.67 | 0.31 | 0.69 | 0.79 (0.76–0.83) |
|  | LR | 0.68 | 0.70 | 0.30 | 0.70 | 0.76 (0.76–0.83) |

**B.**

| **48 hours** | **Models** | **Sensitivity** | **Specificity** | **Brier Score** | **Accuracy** | **AUROC** |
| --- | --- | --- | --- | --- | --- | --- |
| **2015–5019**  **Development and 5-fold cross validation** | XGBoost | 0.64 ± 0.06 | 0.80 ± 0.06 | 0.25 ± 0.04 | 0.75 ± 0.04 | 0.82 ± 0.04 |
|  | RF | 0.73 ± 0.06 | 0.73 ± 0.07 | 0.27 ± 0.06 | 0.73 ± 0.06 | 0.81 ± 0.05 |
|  | LR | 0.68 ± 0.06 | 0.72 ± 0.05 | 0.30 ± 0.02 | 0.70 ± 0.02 | 0.77 ± 0.01 |
| **2020**  **Temporal testing** | XGBoost | 0.65 | 0.81 | 0.23 | 0.77 | 0.82 (0.79–0.86) |
|  | RF | 0.74 | 0.75 | 0.25 | 0.75 | 0.82 (0.81–0.87) |
|  | LR | 0.67 | 0.76 | 0.26 | 0.74 | 0.80 (0.77–0.84) |

AUROC, Area under receiver operating characteristic; LR, logistic regression; RF, random forest; XGBoost, extreme gradient boosting. Values in parentheses are 95% confidence intervals.

**Figure S2.** **Ten most influential features in the XGBoost model over different time horizons (at 24, 48, and 72 hours post-dialysis).** The X-axis and the numbers of the blue bar represent the proportion of each item in the model's prediction.


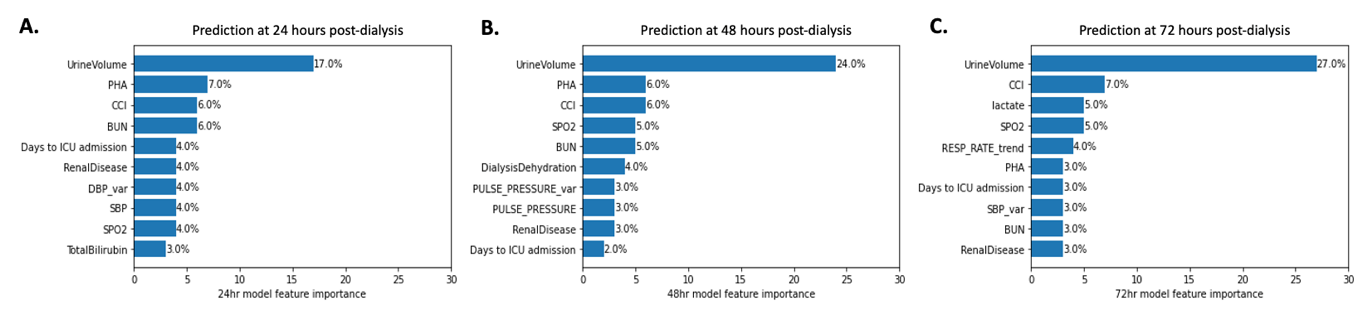


**Figure S3.** (A) 90-day and (B)1-year post-discharge survival for critically ill patients with acute kidney injury requiring dialysis categorized by renal recovery status. The analysis was conducted only for patients who survived to hospital discharge (N = 674).


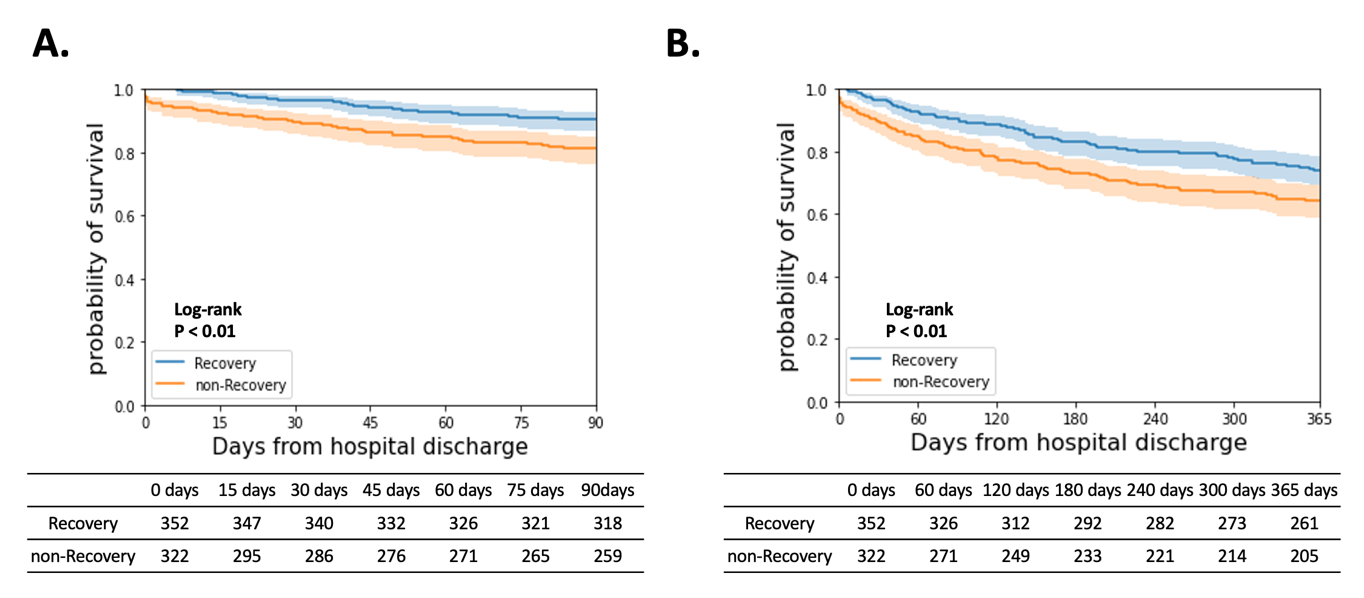
 **Figure S4.** Illustrations of two distinct outcomes at individual patient level. The evolving probability of dialysis liberation and features with highest SHAP scores in the XGBoost algorithm are demonstrated using predictive models at 24, 48, and 72 hours, respectively. (A) Patient with successful dialysis cessation (true positive): The projected likelihood of dialysis cessation incrementally increased in tandem with shifts in the patient's condition, while key predictors also changed. Eventually, the patient became independent of dialysis. (B) For another patient dependent on dialysis (true negative): the anticipated probability of dialysis cessation, as predicted by all three models over different time horizons), remained consistently low and decreased over time. Consequently, ongoing reliance on dialysis was observed.


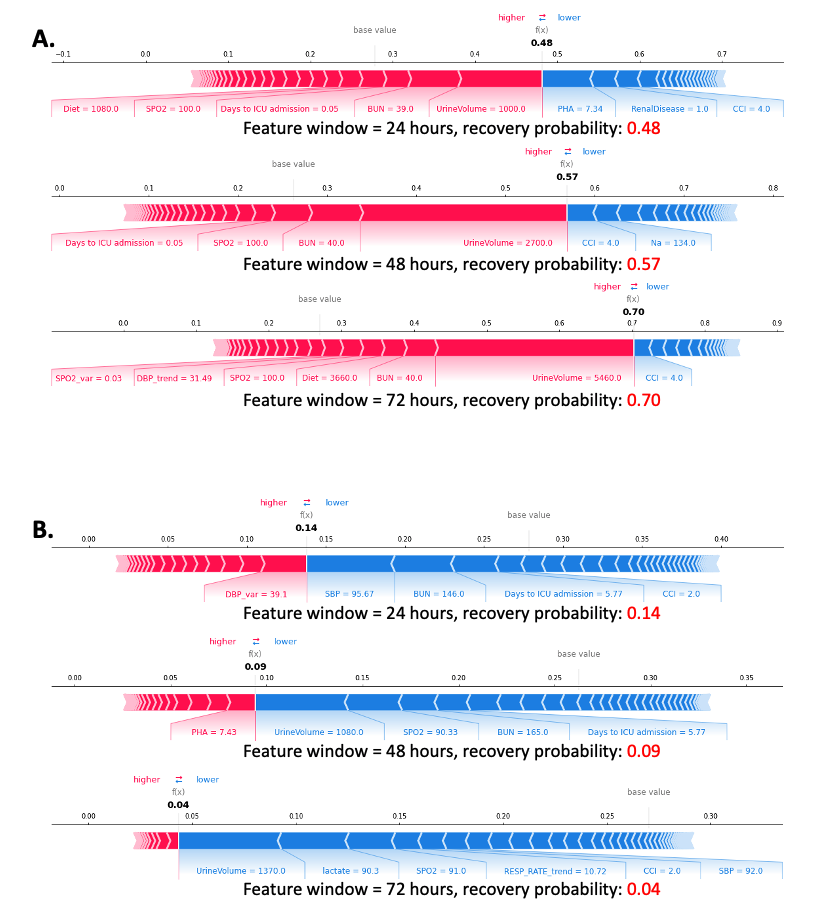

Supplement: Supplementary file 1 — Supplementary Information. [file 41598_2024_63992_MOESM1_ESM.docx]
